# IL-23 inhibitor enhances the effects of PTEN DNA-loaded lipid nanoparticles for metastatic CRPC therapy

**DOI:** 10.3389/fphar.2024.1388613

**Published:** 2024-06-05

**Authors:** Xinlu Chen, Luyao Gong, Yuanyuan Wang, Chen Ye, Huanhuan Guo, Shen Gao, Jiyuan Chen, Zhuo Wang, Yuan Gao

**Affiliations:** ^1^ School of Pharmacy, Fudan University, Shanghai, China; ^2^ Department of Pharmacy, Changhai Hospital, Naval Medical University, Shanghai, China; ^3^ Department of Pharmacy, Shanghai Ninth People’s Hospital, Shanghai Jiao Tong University School of Medicine, Shanghai, China

**Keywords:** prostate cancer, bone metastases, lipid nanoparticles, nucleic acid delivery, immune microenvironment

## Abstract

**Introduction:** Metastatic castration-resistant prostate cancer (mCRPC) patients face challenges due to limited treatment options. About 50% of patients with mCRPC have a functional loss of phosphatase and tensin homology deleted on chromosome 10 (PTEN), leading to tumor progression, metastasis, and immune suppression. Moreover, elevated IL-23 produced by myeloid-derived suppressor cells (MDSCs) is found in CRPC patients, driving tumor progression. Therefore, a combination strategy based on PTEN restoration and IL-23 inhibition may block CRPC progression and metastasis.

**Methods:** The antitumor effect of restoring PTEN expression combined with the IL-23 inhibitor Apilimod was studied in a mouse model of bone metastasis CRPC and mouse prostate cancer RM-1 cells. To verify the targeting ability of PTEN DNA coated with lipid nanoparticles (LNP@PTEN) *in vitro* and *in vivo*. In addition, RT-qPCR and flow cytometry were used to investigate the related mechanisms of the antitumor effect of LNP@PTEN combined with Apilimod.

**Results:** LNPs exhibited significant tumor-targeting and tumor accumulation capabilities both *in vitro* and *in vivo*, enhancing PTEN expression and therapeutic efficacy. Additionally, the combination of LNP@PTEN with the IL-23 inhibitor Apilimod demonstrated enhanced inhibition of tumor growth, invasion, and metastasis (particularly secondary organ metastasis) compared to other groups, and extended the survival of mice to 41 days, providing a degree of bone protection. These effects may be attributed to the PTEN function restoration combined with IL-23 inhibition, which help reverse immune suppression in the tumor microenvironment by reducing MDSCs recruitment and increasing the CD8^+^/CD4^+^ T cell ratio.

**Discussion:** In summary, these findings highlight the potential of LNPs for delivering gene therapeutic agents. And the combination of LNP@PTEN with Apilimod could achieve anti-tumor effects and improve tumor microenvironment. This combinational strategy opens new avenues for the treatment of mCRPC.

## Graphical Abstract

**Figure:**
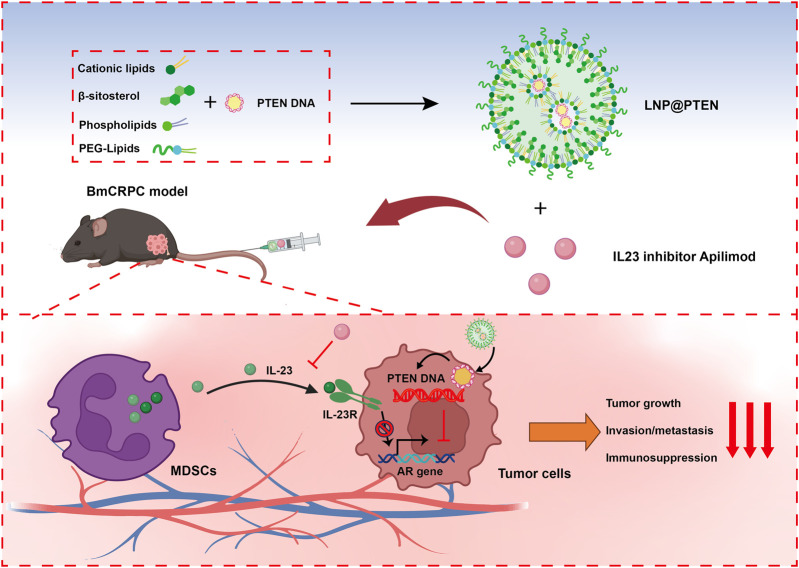
Cationic lipids, β-sitosterol, phospholipids and PEG-lipids self-assemble in aqueous solution to form cationic lipid nanoparticles (LNPs), which could encapsulate PTEN DNA through electrostatic adsorption to generate LNP@PTEN. This formulation could be delivered to prostate cancer bone metastatic sites. LNP@PTEN combined with the IL-23 inhibitor Apilimod showed enhanced anti-tumor effects by inhibiting tumor proliferation and metastasis, and reactivating anti-tumor immunity with good safety (Created with BioRender.com).

## 1 Introduction

Prostate cancer (PCa) is the second most frequent cancer among men worldwide (Sung et al., 2021; Siegel et al., 2023). The emergence of castration-resistant prostate cancer (CRPC) following 18–24 months of androgen deprivation therapy is one of the main clinical challenges in managing PCa (Harris et al., 2009; Jiang et al., 2020). Moreover, a substantial proportion of these CRPC patients progress to a more aggressive stage known as metastatic CRPC (mCRPC), with nearly 90% of cases metastasizing to the bone, leading to the development of bone metastasis CRPC (BmCRPC) with a limited survival period of less than 2 years (Garcia, 2011; Halabi et al., 2016; Chen et al., 2020).

Phosphatase and tensin homology deleted on chromosome 10 (PTEN) loss frequently occurs during human PCa progression. Functional loss of the PTEN tumor suppressor gene is estimated to occur in more than 40% of patients with mCRPC, with up to 70% of advanced-stage samples showing loss of PTEN function (Taylor et al., 2010; Mulholland et al., 2011; Jamaspishvili et al., 2018). The loss of PTEN results in enhanced tumor cell proliferation, viability, and migration, as well as castration-resistant growth (Mulholland et al., 2011). In addition, the loss of PTEN can also promote immune suppression by escalating the population of myeloid-derived suppressor cells (MDSCs) and the secretion of immune-inhibitory cytokines, leading to reduced T-cell infiltration and enhanced infiltration of regulatory T-cells (Treg) within the tumor (Rizvi and Chan, 2016; Bezzi et al., 2018). Therefore, restoring the PTEN function may inhibit the CRPC progression as well as enhance immune function.

Currently, various therapeutic strategies are under exploration to target PTEN-deficient tumors, including conventional inhibition of the PI3K-AKT-mTOR signaling network as well as innovative approaches to restore PTEN function (Mukherjee et al., 2021; Bergholz et al., 2023). *In vitro* experiments have shown that transfecting tumor cells with plasmid DNA can restore PTEN function; however, the use of plasmid DNA still faces challenges related to tumor targeting, transfection efficacy, and maintaining nucleic acid stability (Yin and Anderson, 2017; Islam et al., 2018). Presently, lipid nanoparticles (LNPs) are being used for *in vivo* delivery of nucleic acid drugs (Zhang et al., 2021), which can protect DNA plasmids from enzymatic degradation, prevent unwanted clearance, and promote cellular uptake. Therefore, LNPs could be developed for PTEN DNA delivery, providing an effective therapeutic approach for mCRPC.

In addition, it was reported that patients with CRPC had elevated mRNA levels of IL-23 in the tumor microenvironment, which is released by MDSCs and could activate phoshpo-STAT3–RORγ signaling to drive AR transcription. Therefore, treatments that block IL-23 with its inhibitors may effectively reverse MDSC-mediated resistance to CRPC and synergize with other therapies (Duvallet et al., 2011; Calcinotto et al., 2018).

Due to the high frequency of PTEN loss and high expression of IL-23 in CRPC, the combination of the IL-23 inhibitor Apilimod with PTEN DNA may achieved enhanced effects for cancer therapy. For this purpose, we developed an ionizable cationic lipid, which could self-assemble into LNPs with β-sitosterol, phospholipids, and PEG-lipids for PTEN DNA delivery. We hypothesized that PTEN loaded in LNPs (LNP@PTEN) could effectively reach tumor sites, restoring the anti-tumor function of PTEN. Moreover, the combination of LNP@PTEN with Apilimod could enhance the anticancer effect and immune function for the treatment of CRPC progression.

## 2 Materials and methods

### 2.1 Materials

Commercial suppliers provided all reagents and solvents, which were utilized without additional purification. 1,4-bis (3-aminopropyl) piperazine, 1,2-epoxydodecane, dichloromethane, agarose and crystal violet were acquired from Aladdin (Shanghai, China). 1,2-distearoyl-sn-glycero-3-phosphocholine (DSPC), and 1,2-dimyristoyl-rac-glycero-3-methoxypolyethylene glycol-2000 (PEG_2K_-DMG) were obtained from AVT (Shanghai, China). β-sitosterol was purchased from BIDE (Shanghai, China). Matrigel was obtained from Corning (United States). PTEN plasmid DNA (PTEN), enhanced green fluorescent protein plasmid DNA (pEGFP) and FAM-labeled siRNA (siFAM) were purchased from GenePharma (Shanghai, China). TAE electrophoresis buffer, 4% Paraformaldehyde fix solution, the TUNEL kit, Anti -Ki67 Mouse mAb, and Alexa Fluor^®^ 488-conjugated Goat Anti-Mouse IgG (H + L) were acquired from Servicebio (Wuhan, China). Cell Counting Kit-8 (CCK-8), Propidium iodide (PI), 4′,6-diamidino-2-phenylindole (DAPI) were obtained from Beyotime (Shanghai, China). Apilimod mesylate was purchased from MedChemExpress (United States). Isoflurane was acquired from RWD (Shenzhen, Guangdong). Deoxyribonuclease I (DNase I), Collagenase IV and Hyaluronidase (HAase) were purchased from Biofroxx (Germany). 1,1-dioctadecyl-3,3,3,3-tetramethylindotricarbocyanine iodide (DIR iodide) was obtained from Maokang (Shanghai, China). Distilled water was used throughout the process.

### 2.2 Cell lines and animals

Human embryonic kidney 293T cells (HEK-293T cells), mouse prostate cancer cells (RM-1), mouse embryonic fibroblast cells (NIH-3T3) and mouse embryo osteoblast precursor cells (MC3T3-E1) were provided from Cell Bank of Shanghai, Chinese Academy of Sciences (CAS, Shanghai, China). HEK-293T cells NIH-3T3 cells were cultured in DMEM (Gibco, United States). RM-1 cells were cultured in RPMI-1640 medium (Gibco, United States). MC3T3-E1 cells were cultured in α-MEM medium (Gibco, United States). All cell media were added with 10% fetal bovine serum (FBS) (Gibco, United States) and 100 U/mL penicillin/streptomycin (P/S) (Gibco, United States). The cell culture incubator was used to perform cell culture at a temperature of 37°C and under 5% CO_2_ conditions.

Six-week-old C57BL/6J male normal mice (18–22 g) were ordered from the Clinical Experimental Center, Changhai Hospital, Naval Medical University (Shanghai, China). All experiments were carried out in accordance with the relevant regulations of Committee on Ethics of Medicine, Naval Medical University, PLA. To establish the RM-1 BmCRPC mouse model, RM-1 cells were injected into marrow cavity at 1 × 10^6^ cells per mouse. Tumor formation occurred about 10 d later (Hoang et al., 2017).

### 2.3 Synthesis and identification of cationic lipid 246C10

The 246C10 was synthesized according to the reference (Kim et al., 2021). In detail, in a 5-mL vial, 1 mL of 1,4-Bis (3-aminopropyl) piperazine (4.65 mmol, 4.8 equivalents) was added, and 200 μL of 1,2-Epoxydodecane (0.97 mmol, 1 equivalent) was added dropwise. The reaction mixture was stirred at 90°C with 300 rpm for 3 d, yielding light yellow oil. The product was purified by dichloromethane/methanol column chromatography to obtain high-quality products. The structural identification was performed using hydrogen nuclear magnetic resonance (NMR) and tandem mass spectrometry (MS) spectra.

### 2.4 Preparation and characterization of LNPs

The formulation of LNPs was prepared utilizing the thin-film rehydration methodology. Briefly, 246C10, DSPC, β-sitosterol, and PEG_2K_-DMG were weighed in a molar ratio of 50: 10: 38.5: 1.5 and then were dissolved in anhydrous ethanol, stirring at room temperature (RT) at 100 rpm for 8 h. Subsequently, a thin film was generated through an evaporation process conducted under reduced pressure and then dispersed in pure water using a sonication probe (YM-650Y, Yuming, China) at 400 W for 5 min, resulting in the formation of blank LNPs (LNP-Blank). LNP-Blank was further mixed with PTEN DNA and allowed to incubate at RT for 30 min, ultimately producing PTEN-DNA-loaded LNPs (LNP@PTEN).

The ability of LNPs to transport DNA was assessed via agarose gel electrophoresis. Initially, agarose was dissolved in TAE electrophoresis buffer (0.8% w/v) and heated until completely dissolved. Gel-red (Biosharp, China) was subsequently added to the solution, thoroughly mixed, and then poured into a pre-prepared electrophoresis chamber equipped with a comb. A mixture containing 10 µL of the sample and 1 µL of loading buffer (6×) was prepared and carefully loaded into the wells of the gel after it solidified. Electrophoresis was conducted at RT with 110V for 40 min, and then the gel block was visualized using a UV gel imaging system to capture images.

The morphology of LNP-Blank and LNP@PTEN was assessed using transmission electron microscopy (TEM, TECNAI G2 S-TWIN, United States) after applying negative staining with saturated uranyl acetate solution. Additionally, dynamic light scattering (DLS) analysis was conducted using a Nano ZS90 instrument (Malvern, England) to determine nanoparticles size distribution and zeta potential values. Moreover, to assess their stability in simulated physiological fluids, nanoparticles were stored in PBS (pH 7.4) at 4°C for a period of 20 d.

### 2.5 Gene transfection assays

To evaluate the transfection efficiency of LNPs, HEK-293T cells were seeded in 48-well plates. Subsequently, pEGFP were co-incubated with LNPs at different mass ratios (LNPs: pEGFP = 3, 5, 7, 10), with Lipo8000@pEGFP as the control group (mass ratios is 2). And, pEGFP was 0.5 μg/well. After 24 h of co-culturing with cells, the fluorescence intensity of each group was observed by a fluorescence microscope (CKX53, OLYMPUS, Japan) and quantified by ImageJ software.

### 2.6 *In vitro* cellular toxicity assessment

RM-1 cells and NIH-3T3 cells were cultured in a 96-well plate at a density of 5 × 10^4^ cells/mL. Following this, the cells were co-incubated for 24 h with formulations at varying concentrations (Lipo8000-Blank: 0–50 μg/mL, LNP-Blank: 0–50 μg/mL, PTEN: 1–0.5 μg/mL, Apilomod: 0–10 nM). PBS and NC (PTEN negative control, non-coding random DNA) were used as controls. Cell viability was assessed using CCK-8, and absorbance was measured at 450 nm (OD) by a microplate reader (MULTISKAN MK3, Thermo, United States).

RM-1 cells were seeded in a 48-well plate and cultured overnight. Subsequently, the cells were treated with LNP@pEGFP, with Lipo8000@pEGFP as the control group. After 24 h, RM-1 cells were co-incubated with PI and DAPI (100 μg/mL, 10 μL) in darkness for 30 min and then fixed with paraformaldehyde. Fluorescence expressions within the cells were observed by a fluorescence microscope, and the quantification of fluorescence intensity in each group was conducted using ImageJ software.

### 2.7 *In vitro* cellular uptake and intracellular colocalization assay

To assess cellular uptake efficiency, RM-1 cells were seeded in 24-well plates, then incubated overnight. Subsequently, FAM-labeled siRNA (siFAM) was encapsulated in LNPs and Lipo8000 for 30 min (siFAM, 20 ng/mL). LNP@siFAM was added for co-incubation for 24 h, while an equal volume of PBS, free siFAM and Lipo8000@siFAM served as the controls. Cellular uptake in each group was evaluated via the FACS Calibur flow cytometer (BD Biosciences, United States), with subsequent data analysis employing FlowJo software.

Cover the bottom of the 24-well plate with sterilized coverslips. Following 24 h co-incubation with siFAM, Lipo8000@siFAM and LNP@siFAM, RM-1 cells were fixed with paraformaldehyde, and then a sealing solution containing DAPI was applied to the coverslip. The intracellular distribution of LNP@siFAM was observed by confocal laser scanning microscope (CLSM, SpinSR10, Olympus, Japan).

### 2.8 Lysosome escape assay

To investigate the lysosomal escape capability, RM-1 cells were seeded in 24-well plates, then incubated overnight. Subsequently, LNP@siFAM and Lipo8000@siFAM (siFAM, 20 ng/mL) were co-incubated with RM-1 cells for 1 and 4 h. RM-1 cells were labeled with LysoTracker Red (50 ng/mL) to mark lysosomes, and DAPI was used for cell nucleus staining. Images were acquired by CLSM.

### 2.9 Quantitative real-time PCR

Total RNA was extracted from cellular samples in RNase-free tubes using Trizol (Vazyme, China). Subsequently, reverse transcription into cDNA was carried out with HiScript III All-in-one RT SuperMix Perfect (Vazyme, China). The primer sequences are provided in Supplementary Table S1. Real-time PCR was conducted using Taq Pro universal SYBR qPCR Master Mix (Vazyme, China) following a three-step PCR reaction procedure. Gene expression levels were normalized to GAPDH expression and analyzed using the 2^−ΔΔCt^ method.

### 2.10 *In vitro* cell migration and invasion assays

The *in vitro* inhibitory effects on tumor cell migration and invasion were assessed via Transwell assays. RM-1 cells were cultured in medium without FBS for 24 h. Subsequently, the cells were seeded into the upper chambers of Transwell plates (8-μm, Corning). In the bottom of the chambers, DMEM containing 20% FBS (800 μL) served as chemokines. Matrigel was inserted into the upper chambers of Transwell plates for the anti-migration experiment. Subsequently, RM-1 cells were co-incubated with different treatment groups (PTEN DNA: 0.5 μg/well, Apilimod: 10 nM) for 24 h (anti-migration experiment) and 48 h (anti-invasion experiment). After being fixed in methanol for 30 min, the cells were stained for 20 min with 0.1% crystal violet and then washed three times in PBS (pH 7.2). Bright-field fluorescence microscopy was used to capture images of nine randomly selected fields for each group. Quantitative analysis of cell counts was performed using ImageJ software.

### 2.11 Penetration of LNPs in three-dimensional (3D) multicellular tumor spheroids

The preparation of RM-1 and MC3T3-E1 multicellular spheroids was accomplished through the liquid overlay method. In brief, sterile agarose (50 μL per well) was added to 96-well plates. Subsequently, DiO-stained RM-1 cells and DiD-stained MC3T3-E1 cells (1 × 10^4^ cells each) were proportionally distributed in the 96-well plate at the ratio of 1:1 and then subjected to centrifugation at 1,500 rpm for 12 min at 4°C. During the spheroid formation process, the culture medium (DMEM) was refreshed every 3 d. The image of the tumor spheroids was monitored by CLSM.

Then, 3D multicellular tumor spheroids were prepared without the addition of any dye. LNPs were stained with DiO for 20 min at RT, and then loaded with Cy7-DNA (LNP-DiO@Cy7-DNA, DiO: 20 μg/mL, Cy7-DNA: 0.5 μg/mL), which were co-cultured with the tumor spheroids for 8 h. After fixing with 4% paraformaldehyde, DAPI was employed for nuclei staining. These spheroids were then transferred to a confocal dish, and images were captured by CLSM. Image processing was performed using ImageJ software.

### 2.12 *In vivo* biodistribution study

A mouse model of BmCRPC was established to investigate the *in vivo* distribution of LNPs with DIR serving as a model drug. This was achieved by intravenously injecting free DIR and DIR-loaded LNPs (LNP@DIR, 1 mg/kg). *In vivo* imaging system (IVIS Lumina III *In Vivo* Imaging System, Perkin Elmer) was applied to the *in vivo* fluorescence of each group of mice at 0, 2, 4, 8, 12, and 24 h, as well as fluorescence measurements of all hearts, livers, spleens, lungs, kidneys, and tumor tissues collected from each group of mice euthanized at 24 h. Subsequently, all data were analyzed employing the Quick View 3,000 software.

### 2.13 *In vivo* antitumor effects of LNPs

A mouse model of bone metastasis CRPC was established as previously described. The mice were randomly divided into five groups (*n* = 5): (a) PBS; (b) Apilimod; (c) LNP-Blank; (d) LNP@PTEN; (e) Apilimod + LNP@PTEN (PTEN DNA: 700 μg/kg, Apilimod: 10 mg/kg) (Wada et al., 2006; Wada et al., 2012; Islam et al., 2018). When the tumor size reached approximately 100 mm^3^, the mice received treatment with tail vein injections every 3 days for 2 weeks. Tumor volume and body weight were monitored every other day, with the first dose being reported on the first day. The formula used to compute the tumor volume was V = L × W^2^/2, in which ‘L' stood for the tumor’s longest axis and ‘W' for the axis length that was perpendicular to the longest axis. Sixteen days following the first injection, all animals were anesthetized to obtain blood samples from the retro-orbital venous plexus to assess biochemical indicators (ALT, AST, BUN, and CREAT), and tumor tissues were harvested and weighed. Both tumors and organs were collected to be fixed with 4% paraformaldehyde, embedded in paraffin, and sectioned into tissue slices for subsequent hematoxylin and eosin (H&E) staining examination.

Moreover, five groups of mice (*n* = 5) were randomly divided and treated according to the above protocol for the survival study. According to animal ethical standards, the mice were euthanized when the tumor volume reached 2000 mm^3^.

### 2.14 *In vivo* effects on inhibiting cell proliferation and inducing apoptosis

In the TUNEL staining process, paraffin-embedded sections were deparaffinized in water. Following proteinase K repair, the sections were permeabilized for 10 min at RT. After applying the reaction solution, the sections were incubated for 2 h at 37°C. Lastly, cell nuclei were stained with DAPI by incubating them in the dark at RT for 10 min. Tumor tissue sections were stained by anti-Ki67 mouse mAb (1:200 dilution), followed by an Alexa Fluor 488-labeled goat anti-rabbit IgG (H + L) secondary antibody (1:400 dilution) to assess the proliferation of tumor cells. The cell nuclei were stained with DAPI. The sections were then observed under a fluorescent microscope to capture images. DAPI emits blue light at with an 330–380 nm excitation and 420 nm emission; while TMR emits red light at 520–560 nm excitation and 570–620 nm emission. Alexa Fluor 488 emits green light at 488 nm excitation and 519 nm emission. The ImageJ software was employed to analyze the TUNEL and Ki67 signals in the captured images.

### 2.15 Characterization of the tumor immune microenvironment

The single-cell suspensions of spleens and tumors were prepared as described (Calcinotto et al., 2018; Lin et al., 2021). In brief, tumor tissues underwent mechanical disaggregation followed by enzymatic digestion using collagenase D and DNase for a 30-min incubation at 37°C to achieve a homogeneous single-cell suspension. Each group took three samples and prepared single-cell suspensions in 1.5 mL EP tubes, ensuring a cell count of 10^6^/100 μL PBS per tube using a cell counter. Add 2 uL of Fc Receptor Blocking solution (biolegend, lot no. 422302) to each EP tube, mixed by vortexing, and then placed in the dark for 20 min. Subsequently, single-cell suspensions were labeled with specific monoclonal antibodies, which were primary antibodies directly conjugated. The antibodies used for labeling CD4^+^/CD8^+^ T cells included APC anti-mouse CD45 recombinant antibody (biolegend, clone QA17A26, lot no. 157605), PerCP-Cyanine 5.5 anti-mouse CD4 antibody (biolegend, clone GK1.5, lot no. 100539), and Alexa Fluor 488 anti-mouse CD8a antibody (biolegend, clone 53–6.7, lot no. 100726). The antibodies for labeling MDSCs included FITC anti-mouse CD45 antibody (biolegend, clone I3/2.3, lot no. 147709), APC anti-Ly-6G/Ly-6C (Gr-1) antibody (biolegend, clone RB6-8C5, lot no. 108411), and PE anti-mouse/human CD11b antibody (biolegend, clone M1/70, lot no. 101207). The specific steps were processed in accordance with the manufacturer’s instructions. Then, the samples were incubated in the dark at 4°C for 20 min, centrifuged at 1,000 r/min for 3 min, and the cells were washed with PBS and resuspended in 500 μL PBS. Stained cells were finally evaluated by FACS Calibur flow cytometer (BD Biosciences, United States) and analyzed using FlowJo software.

### 2.16 MicroCT imaging and bone loss analysis

The right hind limb tibia was collected with the normal left hind limb serving as the control (*n* = 3). Bone images were obtained using microCT (μCT-100, SCANCO Medical AG, Switzerland) under 70 kV conditions. The Evaluation V6.5-3 software was utilized for the measurement and analysis of parameters, including bone surface area (BS), total tissue volume (TV), and bone volume (BV).

### 2.17 Statistical analysis

The statistical analysis was carried out with the software Graphpad Prism^®^ 7. In cases where there were just two sample groups, the two-tailed Student’s t-test was utilized. The mean ± SD is used to present the results. When comparing two groups, the Student’s t-test was used; when comparing several groups, one-way analysis of variances (ANOVA) was used. Statistical differences were significant at *p < 0.05 and very significant at **p < 0.01, ***p < 0.001.

## 3 Results

### 3.1 Synthesis and characterization of LNPs

**FIGURE 1:**
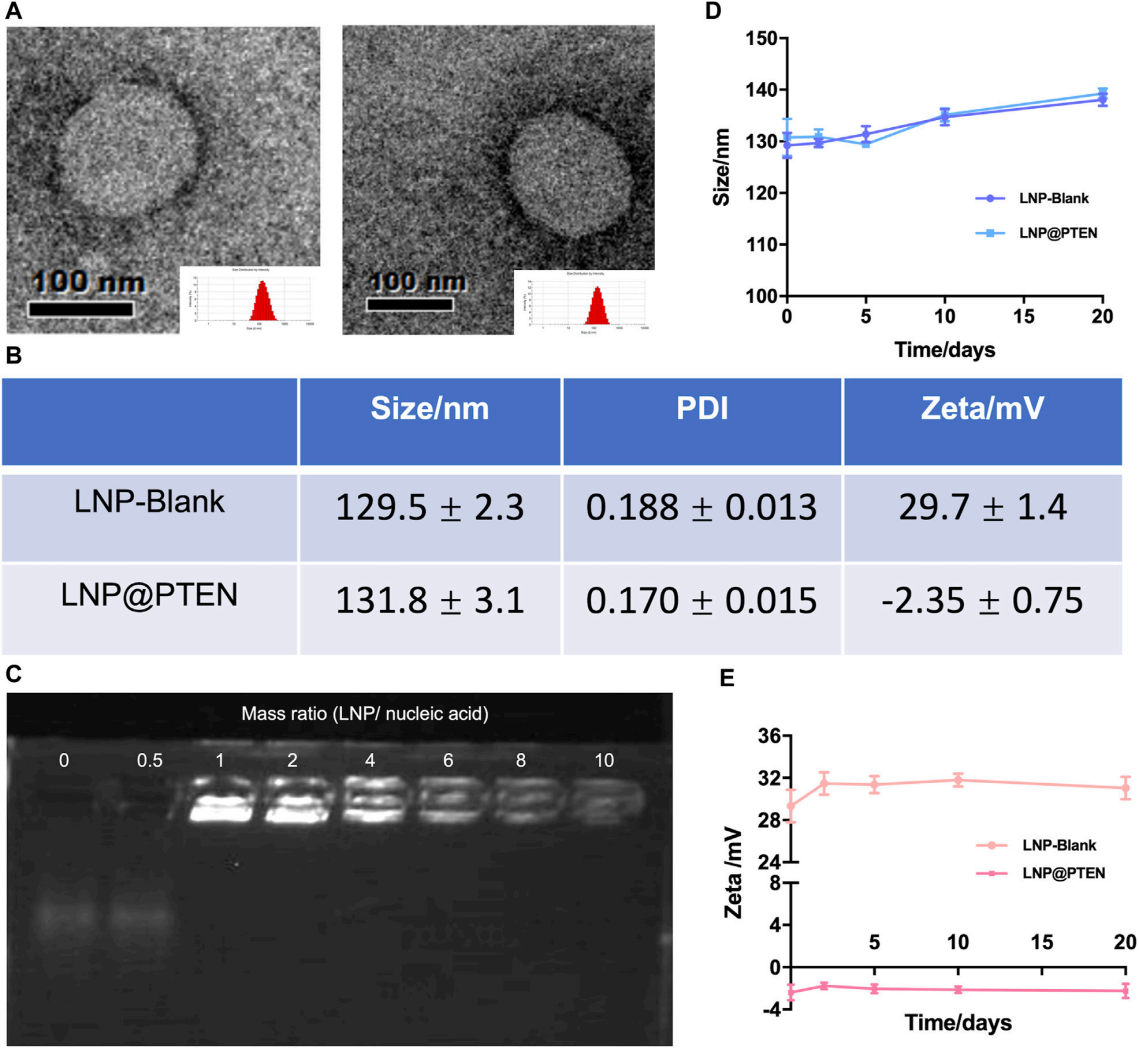
Characterization of LNPs. **(A)** TEM images of LNP-Blank and LNP@PTEN; scale bars = 100 nm. **(B)** The particles size distribution and ζ potential values of LNP-Blank and LNP@PTEN by DLS analysis. **(C)** The agarose gel electrophoresis results of different mass ratios of LNP@PTEN. **(D, E)** The stability of LNP-Blank and LNP@PTEN in PBS (pH 7.4) at 4°C for 20 d (*n* = 3, mean ± SD).

The cationic lipid 246C10, a crucial component of LNPs, was successfully synthesized through the Michael addition reaction, achieving effective gene therapeutic delivery via electrostatic interactions with negatively charged nucleic acids. Detailed synthesis process and characterization of 246C10 are available in the supporting information (Supplementary Figures S1–S3). The formulation of LNPs was composed of 246C10, DSPC, β-sitosterol, and PEG_2K_-DMG. Both LNP-Blank and LNP@PTEN were prepared for characterization. Transmission electron microscopy (TEM) revealed that both LNP-Blank and LNP@PTEN were nanoscale spherical particles with an approximate diameter of 130 nm (Figure 1A, Supplementary Figure S4). The dynamic light scattering (DLS) analysis indicated that the z-average diameter of LNP-Blank was 129.55 ± 2.35 nm, with a polydispersity index (PDI) of 0.188 ± 0.013 (Figure 1B). After co-incubation with the PTEN DNA, the z-average diameter of LNP@PTEN was 131.8 ± 3.1 nm, with a PDI of 0.170 ± 0.015 (Figure 1B). The particle size of the LNPs changed minimally, consistent with that of TEM results. Furthermore, LNP-Blank exhibited positive surface charges, which transitioned to neutral surface charges upon the formation of LNP@PTEN. The ζ-potential values were recorded as (29.7 ± 1.4) mV and (−2.35 ± 0.75) mV, respectively (Figure 1B). When they were incubated in the mimicked physiological fluids of phosphate buffered saline (PBS, pH 7.4) for 20 d, the average diameters and ζ-potential of LNP-Blank and LNP-PTEN were rarely changed (Figures 1D, E). These results confirmed the stability of LNP-Blank and LNP@PTEN in simulated physiological fluids.

Given the enzymatic activity prevalent in the bloodstream, nucleic acids are susceptible to enzymatic degradation, leading to their inactivation. To ensure the effectiveness of gene therapy, LNPs play a pivotal role by encapsulating PTEN DNA, thereby shielding them from enzymatic degradation. We evaluated the encapsulation of PTEN DNA within LNPs using agarose gel electrophoresis (Figure 1C). These findings indicated that when the mass ratio >6, PTEN DNA could be entirely absorbed into the LNPs.

### 3.2 LNPs enhanced cellular uptake and transfection effeciency with lower toxicity *in vitro*

**FIGURE 2:**
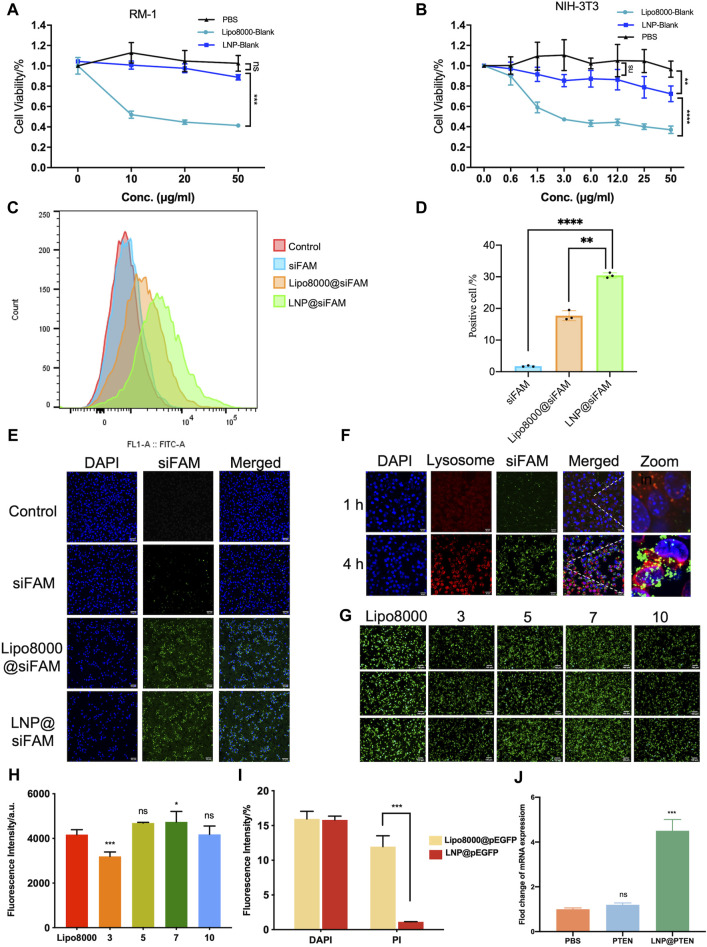
Evaluations of LNPs *in vitro*. **(A, B)** Cytotoxicity of LNPs at different concentrations on RM-1 cells and NIH-3T3 cells for 24 h (*n* = 6, mean ± SD), 2way ANOVA, *p < 0.05, **p < 0.01, ***p < 0.001, ns: no significance. **(C, D)** Statistic results of flow cytometry, RM-1 cells were co-incubated with siFAM, Lipo8000@siFAM, and LNP@siFAM (*n* = 3, mean ± SD), **p < 0.01, ***p < 0.001, one-way ANOVA. **(E, F)** Investigation of intracellular transfection ability in LNPs. CLSM images of **(E, F)** lysosome escape of LNP@siFAM in RM-1 cells for 1 and 4 h scale bars = 20 μm. **(G)** Transfection assessments of LNP@pEGFP complexes on RM-1 cells (*n* = 3). scale bars = 50 μm. **(H)** Fluorescence intensity at different mass ratios in RM-1 cells for 24 h, Lipo8000 was used as control (*n* = 5, mean ± SD), *p < 0.05, **p < 0.01, ***p < 0.001, ns: no significance, one-way ANOVA. **(I)** The fluorescence intensity of DAPI and PI for 24 h after treatment of LNP@pEGFP and Lipo8000@pEGFP (*n* = 3, mean ± SD), ***p < 0.001, ns: no significance, *t*-test. **(J)** RT-qPCR results of the expression of PTEN in RM-1 cells (*n* = 3, mean ± SD), ***p < 0.001, ns: no significance, one-way ANOVA.

The cytotoxicity of LNP-Blank on RM-1 and NIH-3T3 cells for 24 h was investigated using Lipo8000-Blank as a control (Figures 2A, B). Our findings indicated that LNP-Blank has lower cytotoxicity than Lipo8000-Blank on NIH-3T3 cells (*p* < 0.001), with a safety concentration up to 12.5 μg/mL (vs*.* NC group, *p* < 0.05). Similarly, LNP-Blank showed much lower cytotoxicity on RM-1 cells as compared to Lipo8000-Blank (*p* < 0.001), maintains cell viability at approximately 80% at a concentration of 50 μg/mL. These results demonstrated that LNP-Blank had less acute and cell-intrinsic toxicity than Lipo8000-Blank *in vitro*.

Moreover, we investigated the cellular uptake of LNPs in RM-1 cells with siFAM serving as the model drug. Flow cytometry quantification results showed that the cellular uptake of siFAM loaded LNPs (LNP@siFAM) was 1.84 times higher than that of the Lipo8000@siFAM group (Figures 2C, D). The CLSM results showed that green fluorescence (siFAM) was mainly distributed around the cell nucleus (DAPI blue staining). Notably, RM-1 cells exhibited pronounced green fluorescence in LNP@siFAM group, compare to those in free siFAM group (Figure 2E), which was similar to that in the Lipo8000@siFAM group. These results indicated that the LNPs could be effectively internalized by cells, and accurately delivered gene drugs to tumor cells, thus establishing a robust foundation for subsequent drug delivery therapies.

In order to investigate the intracellular transport of LNPs, the lysosomes of RM-1 cells were labeled with LysoTracker Red. After 1 h of incubation, CLSM images illustrated that the red lysosome fluorescence and the green fluorescence (siFAM) overlapped. After 4 h, the green and red fluorescence became clearly separated, indicating that the LNPs had the remarkable lysosomal escape capacity (Figure 2F). Compared with Lipo8000@siFAM (Supplementary Figure S5), both LNPs and Lipo8000 had lysosome escape effect. Consequently, these findings indicated that LNPs can effectively traverse the lysosomal barrier and enhance targeted drug delivery efficiency by efficiently delivering encapsulated drugs to the cytoplasm.

Moreover, we assessed the efficiency of gene transfection in HEK-293T cells *in vitro*. The results demonstrated that a notably higher transfection efficiency was achieved at a mass ratio of 7, surpassing that of Lipo8000 (*p* < 0.05), which indicated that the LNPs had good gene transfection ability and provided the best mass ratio of drug for subsequent assays (Figures 2G, H). In addition, we investigated cell viability post-transfection with LNP@pEGFP and Lipo8000@pEGFP in RM-1 cells with the most optimal transfection efficiency (Figure 2I). PI and DAPI staining were performed on the dead cells and cell nuclei of the two groups, respectively, to assess the proportion of cell death. The findings revealed that the LNP@pEGFP group exhibited superior transfection efficiency compared to that of the Lipo8000@pEGFP group, along with a lower cell death rate (*p* < 0.001), thus underscoring the less acute and cell-intrinsic toxicity of this LNPs *in vitro*.

We further evaluated the impact of LNP@PTEN on PTEN-mRNA levels in RM-1 cells using RT-qPCR (Figure 2J). The results clearly indicated that PTEN-mRNA levels in the LNP@PTEN group were significantly higher than those in both the PBS group and the free PTEN group (*p* < 0.001), affirming the successful delivery of PTEN by LNP@PTEN into tumor cells. These experiments collectively verified the nucleic acid delivery and transfection capabilities of the LNPs *in vitro*.

### 3.3 LNP@PTEN suppressed cell growth, cell invasion and metastasis *in vitro*

**FIGURE 3:**
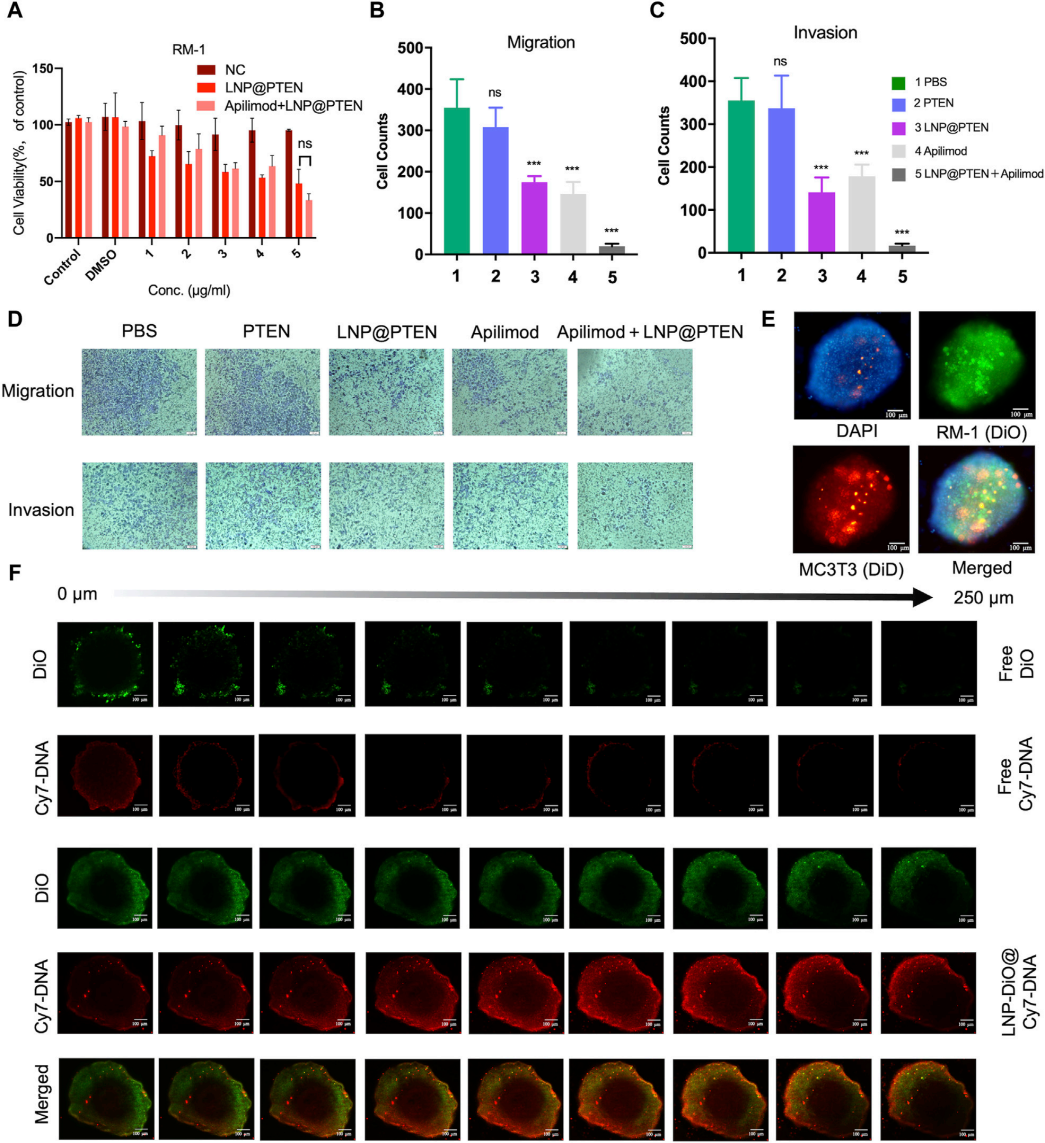
Antitumor assays *in vitro*. **(A)** Cell viability of RM-1 cells after treatment with LNP@PTEN and Apilimod + LNP@PTEN for 24 h (*n* = 3, means ± SD). ns: no significance, 2way ANOVA. **(B, C)** The statistical analysis of cells in nine fields of view of **(B)** anti-migration effects and **(C)** anti-invasion effects, respectively (*n* = 9, mean ± SD), *p < 0.05, **p < 0.01, ***p < 0.001, ns: no significance, one-way ANOVA. **(D)** Microscope images of anti-migration effects and anti-invasion effects in each group (Apilimod: 10 nM, PTEN: 0.5 μg/mL), scale bars = 50 μm. **(E)** 3D tumor spheroid model. DiO-stained RM-1 cells and DiD-stained MC3T3-E1 cells. Blue fluorescence: DAPI, green fluorescence: DiO, red fluorescence: DiD, Scale bars = 100 μm. **(F)** The images of *in vitro* tumor spheroids penetration of each group were acquired by CLSM. DiO-stained LNPs (DiO: 20 μg/mL, Cy7-DNA: 0.5 μg/mL). Scale bars = 100 μm.

Figure 3A showed the results of *in vitro* cytotoxicity of different groups. As shown in Figure 3A, the LNP@PTEN (IC_50_ = 0.4827 μg/mL) group as well as the IL-23 inhibitor Apilimod + LNP@PTEN group (Apilimod/PTEN: IC_50_ = 8.32 nM/0.416 μg/mL) exhibited dose-dependent cytotoxic effects on the RM-1 cells. There was no significant difference between these two groups (*p* < 0.05). Moreover, the Apilimod group showed no significantly higher cytotoxicity than the negative control (NC) group (*p* < 0.05) (Supplementary Figure S6). Our results indicated that there was no obviously exhibition effect of Apilimod on the RM-1 cells *in vitro*. This may due to that the IL-23 was primarily released by MDSCs (Calcinotto A et al., 2018); and the effect of Apilimod might be shown in CRPC microenvironment.

Next, the anti-invasive and anti-metastatic potential of Apilimod + LNP@PTEN was further evaluated by Transwell assay. Microscopic examination of cell migration was conducted. The effects of the combined treatment were subsequently confirmed through quantification of anti-migration and anti-invasion cells (Figures 3B–D). Given the instability of nucleic acids, the cell numbers in the free PTEN DNA group closely resembled those in the PBS group, signifying the absence of any anti-invasive or anti-metastatic influence. In contrast, the LNP@PTEN group and the Apilimod group showed noticeable abilities to inhibit invasion and metastasis in comparison to the PBS group. Furthermore, the combination of LNP@PTEN with the IL-23 inhibitor Apilimod displayed a more pronounced anti-invasive and anti-metastatic ability compared to the monotherapy groups (*p* < 0.001). These results suggested an enhanced effect of the combined treatment in curtailing the invasion and metastasis of RM-1 cells.

### 3.4 LNPs improved penetration in a 3D tumor model

In order to more accurately simulate the *in vitro* bone metastatic microenvironment, we established a 3D tumor spheroid model using MC3T3-E1 and RM-1 cells (Figure 3E). To assess whether LNPs can enhance drug penetration within these spheroids, we loaded Cy7-DNA (red) into LNPs to mimic PTEN DNA, and LNPs labeled with DiO (LNP@DiO, green). Various formulations were applied to treat the 3D tumor spheroids, and the penetration process was visualized using CLSM. Free DiO and free Cy7-DNA were predominantly localized around the spheroids. Importantly, DiO and Cy7-DNA in the LNP-DiO@Cy7-DNA group penetrated the spheroids deeply with the fluorescence intensity much higher than those in the free group at a depth of 250 μm (Figure 3F, Supplementary Figure S7), indicating the superior tumor penetration capability of LNPs.

### 3.5 LNPs efficiently targeted tumor sites

Metastasis is a fatal factor in CRPC progression, and once cancer spreads to the bone, it gives rise to skeletal-related events (SREs), leading to reduced treatment efficacy (Weilbaecher and McCauley, 2011; Steega, 2016). Therefore, we established a mouse model of mCRPC and LNP was labeled with a near-infrared dye (DIR) to investigate the *in vivo* biodistribution of LNPs.

**FIGURE 4:**
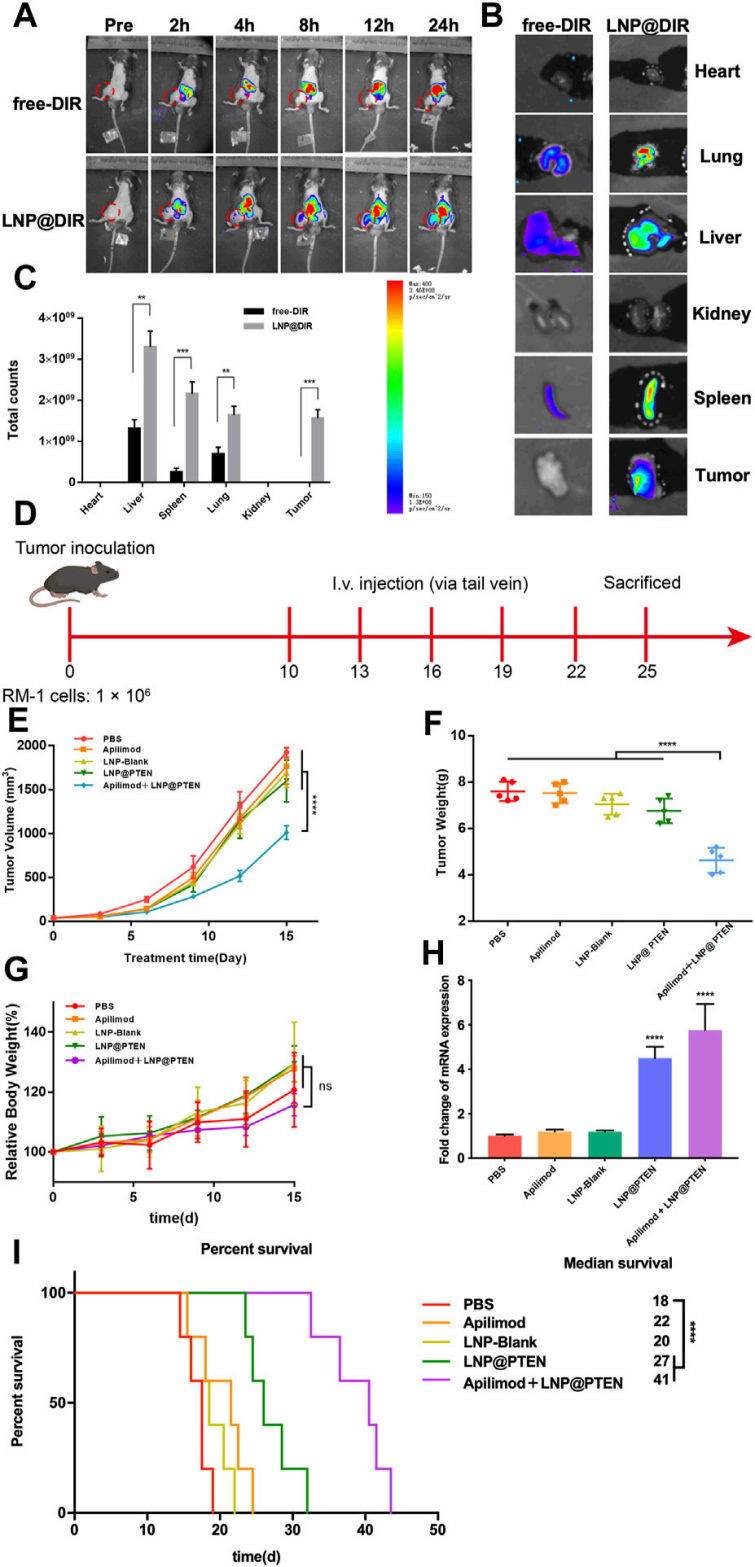
*In vivo* study of LNPs. **(A)** Representative images of BmCRPC-bearing mice from each group were taken at 0–24 h after injection, with the tumor area marked by a red circle. **(B)** Fluorescence imaging of tumors and major organs. **(C)** The semiquantitative fluorescence intensity in major organs of each group (*n* = 3, mean ± SD), *p < 0.05, **p < 0.01, ***p < 0.001, multiple *t*-test. **(D)** The *in vivo* study protocol of Apilimod + LNP@PTEN (PTEN DNA: 700 μg/kg, Apilimod: 10 mg/kg). **(E)** The five groups’ tumor volume curves (*n* = 5, mean ± SD). **(F)** The five groups’ weight curves (*n* = 5, mean ± SD). **(G)** Mice growth curves for body weight among the five groups (*n* = 5, mean ± SD). **(H)** RT-qPCR results of the expression of PTEN in different treatment groups (n = 3, mean ± SD), ***p < 0.001, ****p < 0.0001, one-way ANOVA. **(I)** Survival curve of mice in different treatment groups (*n* = 5, mean ± SD). ****p < 0.0001, ns: no significance, one-way ANOVA.

The *in vivo* biodistribution study results were shown in Figure 4A. As shown in Figure 4A, the fluorescence signal could be monitored at the tumor site in the LNP@DIR group at 2 h early, followed by a gradual increase in the fluorescence signal over the next 16 h, with a peak signal intensity obtained at 24 h in the bone metastasis model. Furthermore, within 24 h, minimal fluorescence was observed at the tumor site in the free DIR group, while substantial DIR accumulated in the liver. The mice were sacrificed after being observed for 24 h in order to measure the biodistribution of fluorescence in different organs. Notably, compared to the free group, the LNP@DIR group exhibited significantly higher fluorescence intensity at the site of the bone metastatic tumor (*p* < 0.001), whereas the fluorescence signal was nearly not detectable in the free DIR group at the primary tumor site (Figures 4B, C). These results substantiated that DIR could be efficiently transported *in vivo*, accumulating at tumor sites via LNPs and achieving targeted delivery to the tumor site through the enhanced permeability and retention (EPR) effect.

### 3.6 LNP@PTEN cooperated with IL-23 inhibitor to inhibit tumor growth and metastasis *in vivo*

We further used BmCRPC models to study the therapeutic effects of combination therapy *in vivo* (Figure 4D). The results showed that the combination of Apilimod + LNP@PTEN group had the strongest antitumor effect among all the groups (Figure 4E). Post-treatment, tumors were subjected to weight measurement (Figure 4F), revealing a significantly reduced tumor weight in the Apilimod + LNP@PTEN group compared to the other treatment groups (*p* < 0.001). This outcome concurred with the tumor volume measurements taken during the course of tumor growth. Furthermore, no substantial variation in mice weight was observed between the Apilimod + LNP@PTEN group and the other treatment groups (*p* > 0.05) (Figure 4G). Remarkably, during the treatment period, mice treated with the five groups displayed analogous and steady weight growth. Moreover, mice treated with Apilimod + LNP@PTEN exhibited a longer median survival time, reaching 40 d in the BmCRPC mouse model (Figure 4I). The q-PCR results showed a significant increase in PTEN mRNA in both the LNP@PTEN group and the combined therapy group (Figure 4H), which indicated that PTEN restoration might contribute to the therapeutic efficacy.

**FIGURE 5:**
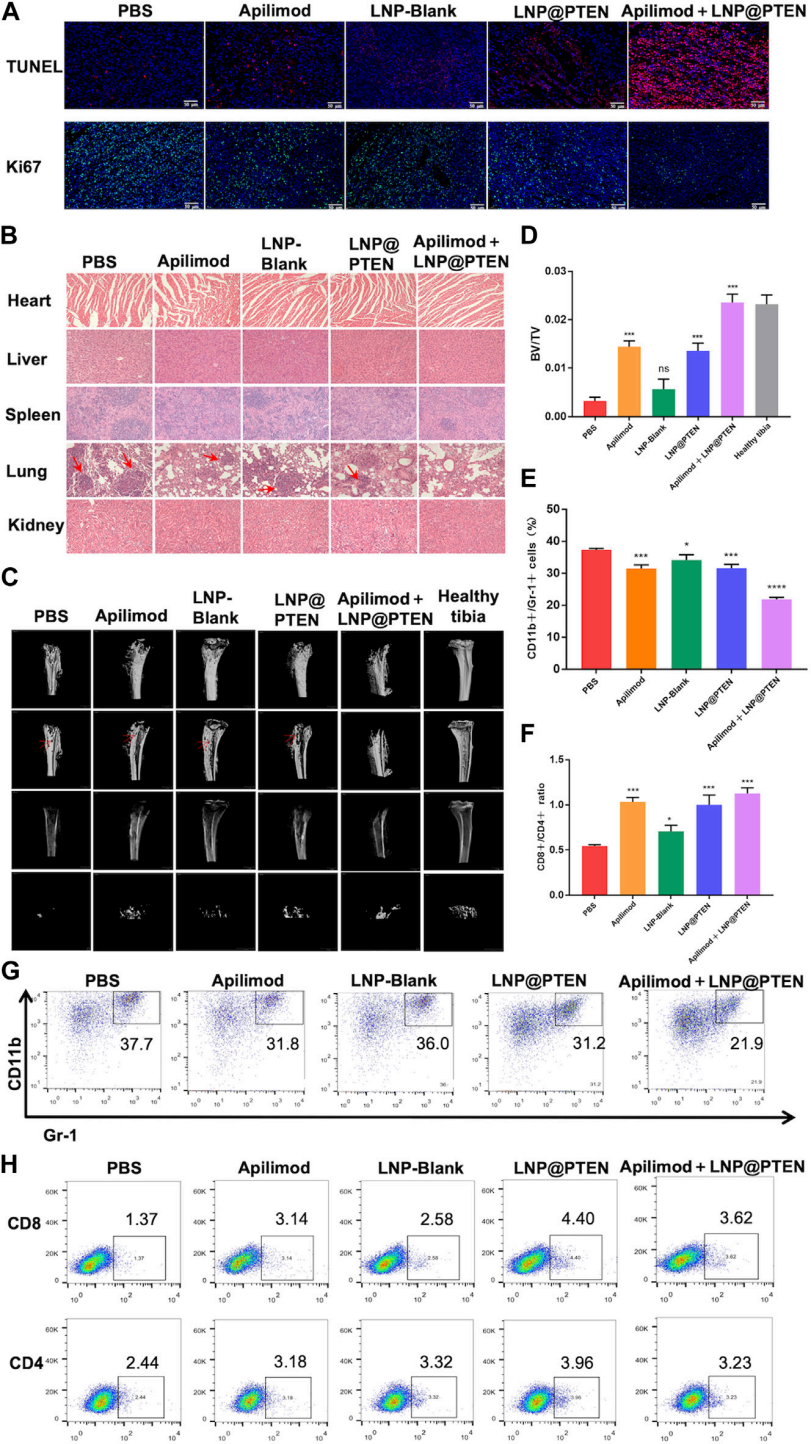
The safety and mechanism of LNPs and immune regulation mechanism. **(A)** TUNEL staining (red) and Ki67 staining (green) of tumor tissues in each group. Scale bars = 50 μm. **(B)** The 200 × HE images of the heart, liver, spleen, lung, kidney of each group (red arrow: Lung metastasis). **(C)** MicroCT images of tibia bearing tumor in each administration group, tibia of normal left hind limb as control (red arrow: Bone erosion and bone damage). **(D)** Tibia BV/TV detection results at different treatment group (*n* = 3, mean ± SD). **(E)** and **(G)** The proportional representative images and statistical images of MDSCs in tumor from various treatment groups (*n* = 3, mean ± SD). **(F, H)** Representative and statistical images of CD8^+^ T cells and CD4^+^ T cells mouse tumor from different treatment groups (*n* = 3, mean ± SD). *p < 0.05, **p < 0.01, ***p < 0.001, ****p < 0.0001, one-way ANOVA.

TUNEL staining was used to detect tumor cell apoptosis. The Apilimod + LNP@PTEN group displayed an elevated number of red fluorescent cells in comparison to the Apilimod and LNP@PTEN groups, signifying a significant induction of cell apoptosis. Notably, no TUNEL-positive cells were observed in the other groups (Figure 5A). Tumor sections were subjected to Ki67 staining, revealing that the Apilimod + LNP@PTEN group exhibited a markedly diminished green fluorescent signal compared to the other groups. These showed the lowest proliferation level of Ki67-positive tumor cells in the combination treatment group, which was consistent with the trends observed in the TUNEL staining (Figure 5A). Collectively, these results provided further compelling evidence of the inhibitory effect of combination therapy on tumor growth.

Additionally, there was no discernible histological damage in all groups when major organs such as the heart, liver, spleen, lung, and kidney were stained with H&E, suggesting that this therapy demonstrated strong tissue tolerance and biocompatibility. (Figure 5B). In line with the previously mentioned findings, the biochemical indicators from mouse blood samples, including serum aspartate aminotransferase (AST), alanine aminotransferase (ALT), blood urea nitrogen (BUN), and creatinine (CR), were demonstrated to be all within the normal range in BmCRPC models after treatment with PBS, Apilimod, LNP-Blank, LNP@PTEN, and Apilimod + LNP@PTEN groups (Supplementary Table S2). These data collectively substantiate the safety profile of the Apilimod + LNP@PTEN combination therapy system. Notably, it is essential to highlight that while tumor metastatic lesions were apparent within the lungs of mice from the PBS group, Apilimod group, LNP-Blank group, and LNP@PTEN group in the bone metastasis model, no secondary organ metastases were discernible in the Apilimod + LNP@PTEN group (Figure 5B).

Subsequently, to assess the potential of Apilimod + LNP@PTEN in SREs associated with BmCRPC, we conducted a comprehensive bone analysis employing microCT (Figure 5C). As a baseline, the left hind limb’s normal tibia in mice served as the control group. In the tumor-bearing tibia of mice from the PBS, Apilimod, LNP-Blank, and LNP@PTEN groups, varying degrees of bone damage and bone resorption were observed, while in the Apilimod + LNP@PTEN group, there was a remarkable mitigation of these effects. Moreover, the bone volume fraction (BV/TV) serves as a widely-used parameter for evaluating cortical and trabecular bone mass. An increase in BV/TV suggests that bone synthesis metabolism surpasses degradation metabolism, leading to an augmented bone mass. This parameter indirectly reflects bone metabolism. Compared to the other treatment groups, the BV/TV ratio in the Apilimod + LNP@PTEN group most closely resembled that of a healthy tibia (Figure 5D). These showed the protective effect of combination therapy on bone metabolism, leading to a reduction in bone damage. These findings substantiated that Apilimod + LNP@PTEN treatment had a significant bone-protective effect. It effectively impeded the growth of BmCRPC tumors, alleviated bone damage and loss in the tumor-bearing tibia, improved SRE outcomes in BmCRPC mice, and proficiently suppressed secondary organ metastasis of BmCRPC.

### 3.7 LNP@PTEN cooperated with IL-23 inhibitor to improve immunosuppressive microenvironment

Bone marrow cell differentiation and functional anomalies represent critical hallmarks of cancer (Croucher et al., 2016). MDSCs, which originate in the bone marrow, play a role in immune suppression and impact T cell activation during the course of cancer development (Kumar et al., 2016). To corroborate the potential of combination therapy in enhancing the tumor immune microenvironment, we employed flow cytometry for the detection of MDSCs (CD11b^+^ Gr-1^+^) infiltration within tumor tissues after 2 weeks treatment (Figures 5E, G, Supplementary Figure S8). The findings revealed that the Apilimod + LNP@PTEN group exhibited the lowest proportion of MDSCs, with a marked reduction compared to the PBS group (*p* < 0.001).

Furthermore, CD4^+^ T cells fulfill a regulatory role in immune responses, while CD8^+^ T cells serve as vital effector cells in the context of anti-tumor responses, and an elevated CD8^+^ ratio is indicative of a more potent anti-tumor effect (Shi et al., 1995). The outcomes demonstrated that the Apilimod + LNP@PTEN group attained the highest CD8^+^/CD4^+^ ratio (Figures 5F, H, Supplementary Figure S9), implying a more robust anti-tumor immune response relative to the other treatment groups. This heightened immune response played as a pivotal contributor to the effective inhibition of CRPC development in the combination therapy group.

## 4 Discussion

The loss of the tumor suppressor PTEN is a common occurrence in CRPC. Up to 40% of patients with mCRPC face the challenge of PTEN deficiency. In addition, PTEN insufficiency can also promote immune suppression by escalating the population of MDSCs and the secretion of the immune-inhibitory cytokines IL-23 (Calcinotto et al., 2018). Therefore, restoration of PTEN function as well as inhibiting IL-23 to reverse MDSC-mediated resistance to CRPC may bring potential therapies for mCRPC treatment.

Promising therapeutic strategies have been developed to restore PTEN function alone or combine with PI3K-AKT-mTOR inhibition, chemotherapy, androgen receptor-directed agents, DNA damage response (DDR) -targeting agents, immune oncology agents, or additional PI3K-AKT-mTOR-targeted therapies (Turnham et al., 2020). Among these approaches, gene therapy is most effective in restoring PTEN function. However, these strategies face challenges such as low delivery efficiency, poor transfection efficiency, insufficient expression, and the potential for insertional mutations (Islam et al., 2018). Therefore, novel gene vectors with high efficiency and low toxicity still need to be further explored.

Here, in our study, we successfully developed an ionizable cationic lipid 246C10 that could self-assemble into LNPs with β-sitosterol, phospholipids, and PEG-lipids for PTEN DNA delivery. The characterization results indicated that LNP-Blank possesses a positive charge of 29.7 mV and maintains a particle size of approximately 130 nm. Following the loading of PTEN DNA, the particle size increased slightly, and the charge became neutral (Figures 1A, B). The *in vitro* and *in vivo* results demonstrated the high transfection efficiency of LNPs and low toxicity of the LNP-Blank (Figures 2A, B, and Supplementary Table S2), which are suitable as carriers for PTEN DNA delivery.

Previous study reported that elevated levels of MDSCs and IL-23 were found in the blood and tumor samples of CRPC mice and patients, compared to those in hormone-sensitive PCa patients (Calcinotto et al., 2018). Our results showed that IL-23 inhibitor Apilimod alone had no obvious effect on RM-1 cells *in vitro* (Supplementary Figure S6; Figure 3A) and *in vivo* (Figure 4E), while significantly improved the anticancer effect of LNP@PTEN *in vivo* (*p* < 0.001) (Figure 4E). This demonstrated that the IL-23 was mainly released by MDSCs, which was in line with the result reported (Calcinotto et al., 2018). Moreover, the combination of LNP@PTEN with IL-23 inhibitor Apilimod showed a significantly anticancer effect (Figure 4E). All these results indicated that for PTEN-deficient CRPC patients, only restoration of PTEN function may be not enough to inhibit the CRPC progression, and the combination therapies with other strategies are needed. Furthermore, the MDSCs in tumor microenvironment play a critical role for CRPC progress, and blocking MDSCs recruiting is one of the choices for combinational therapies. Therefore, the combination of LNP@PTEN and Apilimod may provide a potential strategy for CRPC clinical treatment, especially for PTEN-deficient CRPC patients.

Since 90% of patients with CRPC will eventually develop metastasis to bone. Once cancer spreads to the bone, it gives rise to SREs, such as severe pain and abnormal bone remodeling, leading to poor prognosis and reduced treatment efficacy (Weilbaecher and McCauley, 2011; Steega, 2016). We established a CRPC bone metastasis animal model and conducted an intra-tumoral distribution study using LNPs in these model mice. The *in vivo* distribution experiments indicated the successful accumulation of LNPs (LNP@DIR) in the tumor sites of tumor-bearing mice, which may be associated with the nanoparticle’s EPR effect (Figures 4A–C). Moreover, our research demonstrated the highest effectiveness of the Apilimod + LNP@PTEN among all groups, showing superior anti-tumor growth, anti-invasive and anti-metastatic properties as compared to those of monotherapy *in vivo* (Figures 4E–I). In addition, we also found that the combination therapy of LNP@PTEN and Apilimod also significantly inhibited bone metastasis CRPC distant organ secondary metastasis with excellent safety (Figures 4G, 5B; Supplementary Table S2).

Furthermore, we investigated the immunomodulatory effects of the combination therapy by assessing immune cells in tumor tissues, thereby alleviating immune suppression, enhancing the immune response, and inhibiting tumor progression to some extent (Figures 5E–H). The PTEN/PI3K/AKT pathway had been shown to be a crucial route for MDSCs activation, and MDSCs indirectly promote the development of CRPC through the secretion of IL-23. LNP@PTEN compensated for PTEN deficiency, blocking the activation of this pathway in MDSCs, thereby reducing the proportion of immunosuppressive cells. Additionally, the reduction in MDSC activation and interference with IL-23’s regulatory role in tumor development, due to the application of the IL-23 inhibitor, relieved immune suppression from two different aspects. This is probably a key reason for the inhibition of tumor growth in the Apilimod + LNP@PTEN group. Finally, we also investigated the bone-protective effects of the treatment. The combined intervention of LNP@PTEN + Apilimod could maximize the preservation of normal bone metabolism, reducing bone injury and resorption (Figures 5C, D).

## 5 Conclusion

In summary, a combinational therapeutic strategy based on LNP@PTEN and the IL-23 inhibitor Apilimod was established, which could significantly inhibit tumor growth and metastasis for CRPC. The prepared LNPs exhibit excellent stability and high transfection efficiency, allowing for efficient drug delivery to tumor sites. Furthermore, the combination of LNP@PTEN and Apilimod inhibited tumor progression, distant organ secondary metastases, and extended the survival period of tumor-bearing mice, by the restoration of PTEN function and reversing the immunosuppressive tumor environment. This work provides a new strategy for CRPC treatment.

## Data Availability

The original contributions presented in the study are included in the article/supplementary material, further inquiries can be directed to the corresponding authors.
