# Supplementary material for: IL-23 inhibitor enhances the effects of PTEN DNA-loaded lipid nanoparticles for metastatic CRPC therapy
Source: Front Pharmacol. 2024 Jun 5;15:1388613. doi: 10.3389/fphar.2024.1388613 (PMC11186457; doi:10.3389/fphar.2024.1388613)
Supplement: Supplementary file 1 [file DataSheet1.docx]

Supplementary Material

# 1 Supplementary Figures and Tables

**Supplementary Figure S1.** Detailed synthesis procedure of 246C10, which was divided in one step (Kim M, 2021).

**Supplementary Figure S2.** MS analysis of 246C10.

**Supplementary Figure S3.** Characterization of 246C10 by ^1^H NMR spectrum.

**Supplementary Figure S4.** TEM images of (A) LNP-Blank and (B) LNP@PTEN; scale bars = 100 nm.

**Supplementary Figure S5.** Lysosome escape of Lipo8000@siFAM in RM-1 cells for 1 h and 4 h. scale bars = 20 μm.

**Supplementary Figure S6.** Apilimod inhibited RM-1 cell proliferation. (Apilimod: 0~10 nM), (n = 3, mean ± SD).ns: no significance, 2way ANOVA.

**Supplementary Figure S7.** Mean fluorescence intensity of 3D tumor spheroids penetration of each group (Free-DiO, Free Cy7-DNA, and LNP-DiO@Cy7-DNA) (n = 3, mean ± SD). **p* < 0.05, ***p* < 0.01, ****p* < 0.001, ns: no significance, one-way ANOVA.

**Supplementary Figure S8.** The contour plot of MDSCs in tumor from various treatment groups.

**Supplementary Figure S9.** The contour plot of CD8^+^ and CD4^+^ T cells in tumor from various treatment groups.

**Supplementary Table S1.** primer sequence

**Supplementary Table S2.** Biochemical analysis of mouse blood after 2 weeks of treatment

ALT, Alanine aminotransferase; AST, Aspartate aminotransferase; BUN, Urea nitrogen; CR, Creatinine. Data represent the mean ± SD (n = 3 mice per group).

PTEN Coding Sequence(CDS):

ATGACAGCCATCATCAAAGAGATCGTTAGCAGAAACAAAAGGAGATATCAAGAGGATGGATTCGACTTAGACTTGACCTATATTTATCCAAACATTATTGCTATGGGATTTCCTGCAGAAAGACTTGAAGGCGTATACAGGAACAATATTGATGATGTAGTAAGGTTTTTGGATTCAAAGCATAAAAACCATTACAAGATATACAATCTTTGTGCTGAAAGACATTATGACACCGCCAAATTTAATTGCAGAGTTGCACAATATCCTTTTGAAGACCATAACCCACCACAGCTAGAACTTATCAAACCCTTTTGTGAAGATCTTGACCAATGGCTAAGTGAAGATGACAATCATGTTGCAGCAATTCACTGTAAAGCTGGAAAGGGACGAACTGGTGTAATGATATGTGCATATTTATTACATCGGGGCAAATTTTTAAAGGCACAAGAGGCCCTAGATTTCTATGGGGAAGTAAGGACCAGAGACAAAAAGGGAGTAACTATTCCCAGTCAGAGGCGCTATGTGTATTATTATAGCTACCTGTTAAAGAATCATCTGGATTATAGACCAGTGGCACTGTTGTTTCACAAGATGATGTTTGAAACTATTCCAATGTTCAGTGGCGGAACTTGCAATCCTCAGTTTGTGGTCTGCCAGCTAAAGGTGAAGATATATTCCTCCAATTCAGGACCCACACGACGGGAAGACAAGTTCATGTACTTTGAGTTCCCTCAGCCGTTACCTGTGTGTGGTGATATCAAAGTAGAGTTCTTCCACAAACAGAACAAGATGCTAAAAAAGGACAAAATGTTTCACTTTTGGGTAAATACATTCTTCATACCAGGACCAGAGGAAACCTCAGAAAAAGTAGAAAATGGAAGTCTATGTGATCAAGAAATCGATAGCATTTGCAGTATAGAGCGTGCAGATAATGACAAGGAATATCTAGTACTTACTTTAACAAAAAATGATCTTGACAAAGCAAATAAAGACAAAGCCAACCGATACTTTTCTCCAAATTTTAAGGTGAAGCTGTACTTCACAAAAACAGTAGAGGAGCCGTCAAATCCAGAGGCTAGCAGTTCAACTTCTGTAACACCAGATGTTAGTGACAATGAACCTGATCATTATAGATATTCTGACACCACTGACTCTGATCCAGAGAATGAACCTTTTGATGAAGATCAGCATACACAAATTACAAAAGTCTGA

## References

[1] Kim M, J. M., Hur S, Cho Y, Park J, Jung H, Seo Y, Woo HA, Nam KT, Lee K, Lee H. (2021). Engineered ionizable lipid nanoparticles for targeted delivery of RNA therapeutics into different types of cells in the liver. Sci Adv. 7(9), eabf4398. doi:10.1126/sciadv.abf4398.
